# A 3-Year Split-Mouth Randomized Controlled Clinical Trial of Zirconia and Titanium Implant-Supported Overdentures

**DOI:** 10.3390/jfb17050213

**Published:** 2026-05-01

**Authors:** Kristian Kniha, Lothar Rink, Mark Ooms, Katharina Schaffrath, Stephan Christian Möhlhenrich, Frank Hölzle, Ali Modabber, Marius Heitzer

**Affiliations:** 1 Department of Oral and Cranio-Maxillofacial Surgery, University Hospital RWTH Aachen, Pauwelsstraße 30, 52074 Aachen, Germany; 2 Private Dental Clinic, Dres. Kniha, Rosental 6, 80331 Munich, Germany; 3 Institute of Immunology, University Hospital RWTH Aachen, Pauwelstraße 30, 52074 Aachen, Germany; 4 Department of Dentistry, Section of Orthodontics, Dentofacial Orthopedics and Craniofacial Biology, Radboud University Medical Centre, THK 309, P.O. Box 9101, 6500 HB Nijmegen, The Netherlands

**Keywords:** dental implant, ceramic, zirconium oxide, titanium, removable denture, cytokine

## Abstract

Aim: This study aimed to compare two-piece zirconia and two-piece titanium implants inserted into the anterior mandible for removable overdentures in a 3-year randomized split-mouth clinical trial. Methods: Twenty fully edentulous mandibular patients received two zirconia and two titanium implants allocated by computer-generated randomization. The primary endpoint was bleeding-on-probing (BOP) at 12 months. Secondary outcomes included implant survival and success (Albrektsson criteria), marginal bone level changes, peri-implant cytokines (IL-1β, IL-6, and TNFα), prosthetic complications, and patient-reported outcomes (PROMs). Results: After 3 years, overall survival was 98.61% and overall success was 84.72%. Titanium implants showed higher success compared with zirconia implants (91.70% vs. 77.78%), while survival was 100% and 97.22%, respectively. Marginal bone loss was significantly greater around zirconia implants at 36 months (*p* < 0.01). No significant differences were observed in IL-1β, IL-6, or TNFα levels up to 12 months. PROMs revealed a trade-off, with zirconia favored for esthetics and cleaning perception, while titanium was rated superior for stability. Conclusions: Within the limitations of this split-mouth RCT, zirconia implants demonstrated reduced success and inferior marginal bone stability compared with titanium implants in overdenture therapy. Careful case selection and close follow-up appear essential when zirconia implants are used in this indication.

## 1. Introduction

Dental implants are a well-established and predictable modality for the functional and esthetic rehabilitation of tooth loss. Titanium implants continue to be regarded as the gold standard owing to their superior biocompatibility, reliable osseointegration, and consistently high long-term survival rates [1].

Complications of dental implants include peri-implant inflammation, which may occur on a daily basis. Current data show that mucositis tends to develop in 43% of implants, leading to peri-implantitis in 22% of cases [2]. Thus, the development of dental implant materials that alleviate the risk of inflammation remains desirable. Zirconia oxide ceramics have provided new zirconia implant alternative options for dental implantology and have shown superior biomechanical properties, such as high fracture toughness and bending strength, compared to other ceramics [3]. For these reasons, zirconia is currently the material of choice for the fabrication of ceramic dental implants [4]. Furthermore, laboratory investigations have shown that zirconia implants, such as those using magnesium, aluminum, or yttrium ions, are very reliable products [5], with the additional benefit of potentially avoiding the risk of implant fractures [6].

A review of the available literature indicates that one-piece zirconia implants achieve osseointegration similar to that of titanium implants, with stable crestal bone [7]. Furthermore, immediate loading or temporization of the implants does not interfere with the course of osseointegration. However, scientific evidence supporting the use of two-piece implants is rare [7]. Overall, the biological healing responses induced by ceramic surfaces have been rated as more desirable than those achieved using metal surfaces [8]. Peri-implant soft and hard tissue behaviors have been deemed very favorable [9], with ceramics providing a superior formation of the epithelial attachments and mucosal conditions [10] considered important for preventing peri-implant infections.

The hope that new dental implant products will reduce the incidence of peri-implant infections is supported by microbiological and clinical studies demonstrating a much lower plaque affinity with ceramic materials than with titanium [11,12]. Indeed, modern zirconia implants with a microrough surface topography might be potential alternatives to titanium for the fabrication of dental implants. Consequently, further science-based clinical results are needed for commercially available zirconia dental implants, but few clinical investigations have been published, especially for removable dentures consisting of two-piece zirconia implants.

The primary aim of the present study was to evaluate the improvement in a clinical parameter (bleeding-on-probing index) for zirconia implants over a one-year period. Specifically, the purpose was to compare two-piece zirconia and two-piece titanium implants inserted into the anterior mandibula as removable overdentures. The working hypothesis was that a lower proinflammatory parameter leads to less soft-tissue inflammation (at the time of overdenture insertion and at 6, 12, and 36 months after insertion), assessed by the bleeding-on-probing index around implants. Peri-implant bone loss and proinflammatory parameters were also assessed.

## 2. Materials and Methods

In this prospective split-mouth clinical trial, 20 fully edentulous participants in the mandible were enrolled. Following a standard two-stage surgical protocol involving initial unloaded healing and subsequent loading after three months, each patient received four implants in the interforaminal region: two zirconia implants (Pure Ceramic CI, 4.1 mm diameter, Straumann GmbH, Freiburg, Germany) and two titanium implants (TL SLActive, 4.1 mm diameter, Institut Straumann AG, Basel, Switzerland). Randomization was performed using a computer-generated block randomization sequence with a 1:1 allocation ratio to determine the assignment of zirconia and titanium implants to the left and right interforaminal regions. The randomization list was generated by an independent investigator who was not involved in the surgical procedures or outcome assessment. Allocation was concealed using sequentially numbered, opaque, sealed envelopes that were opened immediately prior to implant placement. This procedure ensured that the operating surgeon was unaware of the implant material allocation until the time of surgery, thereby minimizing selection bias. Due to the visible differences between implant materials, blinding of the surgeon and clinical examiner during follow-up was not feasible.

All implants, placed by a single surgeon in accordance with the manufacturer’s guidelines, had a diameter of 4.1 mm and a length ranging from 8 to 12 mm. Overdentures were retained using Novaloc or Pureloc locator attachments (Institut Straumann AG, Basel, Switzerland). In the zirconia group, two-piece zirconia implants were restored using zirconia locator abutments (Pureloc system). The abutments were secured to the implant bodies with a titanium fixation screw, as specified by the manufacturer. In the titanium group, titanium implants were restored with corresponding titanium locator abutments and titanium components throughout the restorative chain.

**Figure 1:**
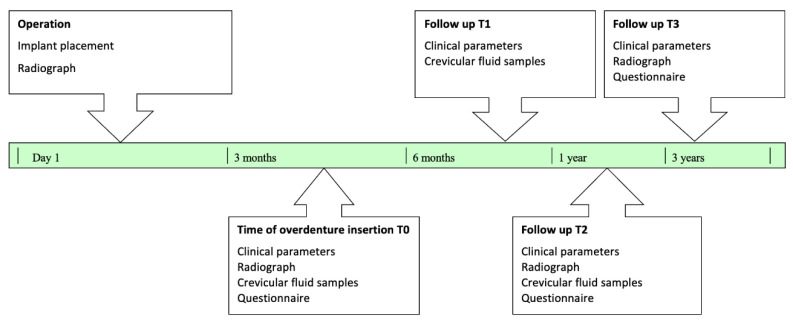
Timeline of the three-year follow-up protocol.

Inclusion criteria were fully edentulous mandibles and adequate bone volume for all implants. Exclusion criteria included systemic disease (e.g., uncontrolled diabetes), smoking, untreated periodontitis or gingivitis, and severe bruxism or clenching. Participants were followed for a 36-month period, during which bacterial samples were collected at 3 months (T0), 6 months (T1), 12 months (T2), and 36 months (T3) (Figure 1). Throughout the study, the patients were instructed to maintain optimal oral hygiene. Prior to sampling, they were required to refrain from rinsing, antibiotic use, or food intake.

**Figure 2:**
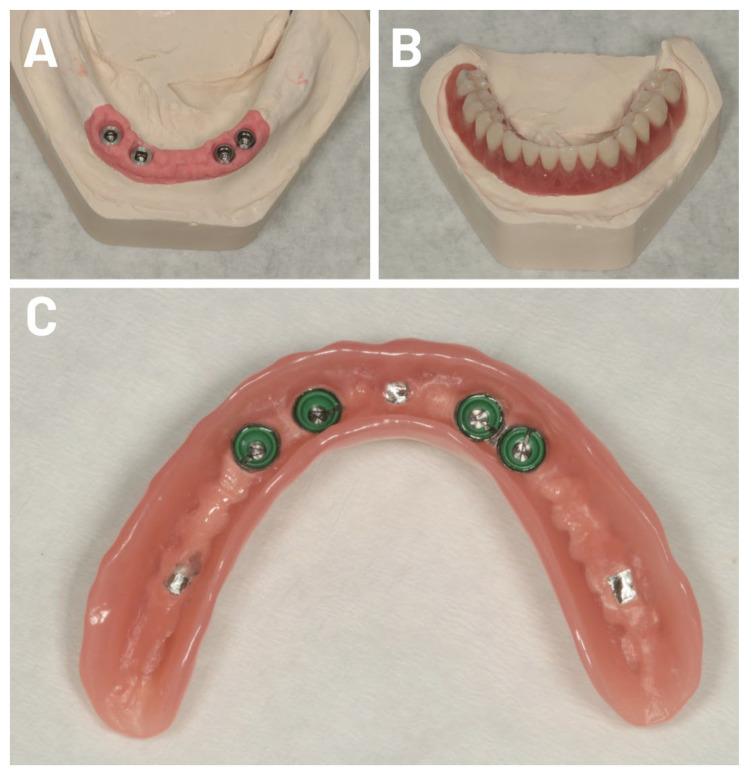
(**A**) The master cast for one representative case (Figure 2). (**B**) The dental lab returned the finalized Novaloc^®^ overdenture to the dental office, with the included mounting inserts in place. Novaloc^®^ retention inserts were selected and inserted. (**C**) Finally, the finished overdenture was seated.

**Figure 3:**
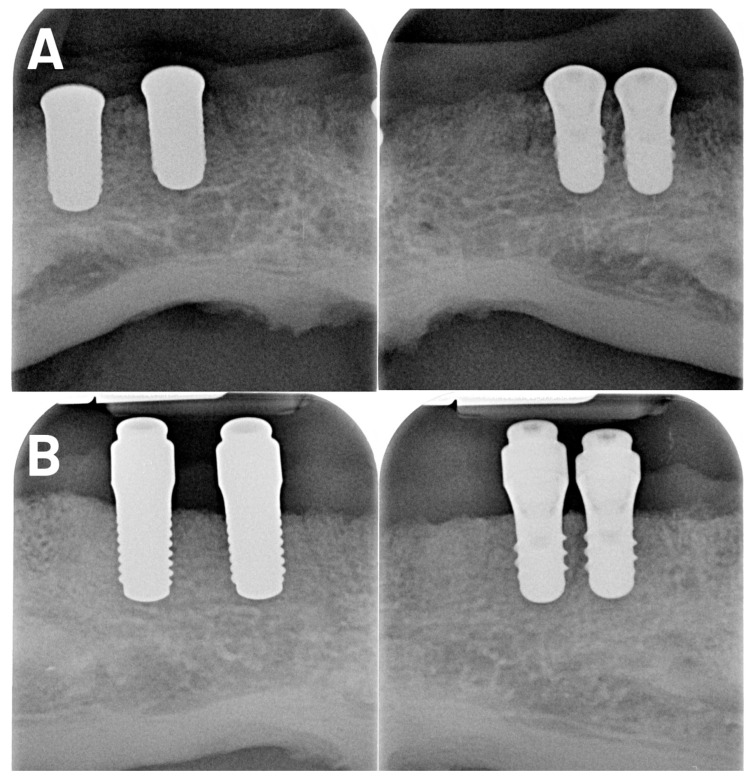
Radiologic presentation of a follow-up case. Radiologic control images show the zirconia implants and titanium implants ((**A**) CI left and TI right). Radiological images were also taken at 36 months after insertion ((**B**) CI left and TI right).

**Figure 4:**
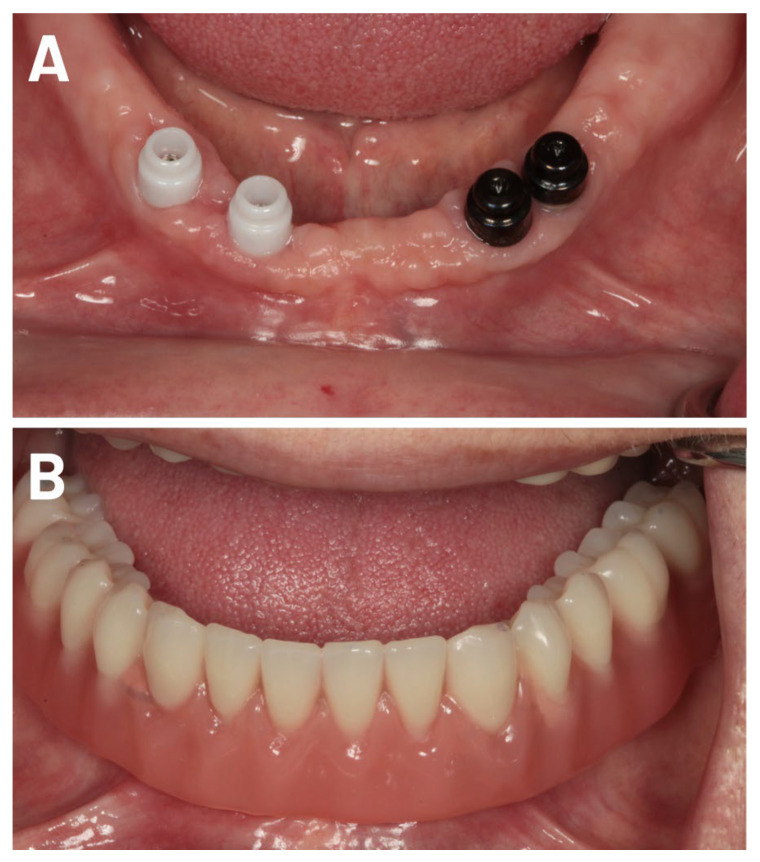
(**A**) Final photo of the implant locators at the 3-year follow-up. (**B**) View of the inserted removable denture at the 3-year follow-up.

After the healing period, impressions of the jaw (Alginat, Müller-Omicron GmbH & Co. KG, Lindlar, Germany) were taken to produce individual impression trays. The overdentures were fabricated following the individual open-impression technique. Novaloc^®^ abutments were inserted using the mucodynamic technique for impression taking (vinyl polysiloxane or polyether rubber), and the master cast was then fabricated (Figure 2, Figure 3 and Figure 4). The dental lab returned the finalized Novaloc^®^ overdenture, with the mounting inserts in place, to the dental office. Novaloc^®^ retention inserts were selected and inserted, and the finished overdenture was seated.

This study was designed as a randomized controlled clinical trial (split-mouth design) in which two CE-marked dental implant systems were evaluated within the scope of their intended use. In accordance with the applicable legal framework of the EU Medical Device Regulation (MDR), the study is classified as a clinical investigation of medical devices in routine clinical use and was not subject to mandatory prospective registration in a public clinical trials registry. This clinical trial was registered in the German Clinical Trial Register (DRKS; registration number DRKS00039113). Registration was performed retrospectively after study initiation to meet publication requirements. Recruitment started in 2019, and the final 3-year follow-up was completed in 2024. No changes were made to the study design, protocol, or predefined endpoints after study initiation or during the course of the study.

### 2.1. Experiment Protocol

Variables were the implant survival and success rates, which were compared for the zirconia and titanium implants. One calibrated investigator performed all the measurements. All the patients were examined clinically and radiographically on the date of implant placement and after 3 years using a measurement method analogous to a previously published one [13].

The success rate was determined using the method described by Albrektsson et al. [14], who used a combination of anamnestic data, clinical examination results, and radiological findings to categorize the success rates of implants based on the following assessments: no pain or discomfort, immobility, absence of radiolucency, and bone resorption of less than 0.2 mm per year from the time point of implant loading. Peri-implant tissue health was measured at 0 (T0), 6 (T1), 12 (T2), and 36 (T3) months after insertion using the gingiva index (GI, Silness & Löe, score 0 = normal gingiva, 1 = mild inflammation, 2 = moderate inflammation, and 3 = severe inflammation), plaque index (PlI, Silness & Löe, score 0 = no plaque, 1 = plaque not visible but verified with a probe, 2 = visible plaque, and 3 = massive plaque), both adapted for dental implants (mGI + mPlI; [15]), and the bleeding-on-probing index (BOP, Saxer & Mühlemann, score 0 = no bleeding, 1 = isolated bleeding, 2 = confluent linear bleeding, and 3 = severe bleeding) [15,16,17]. The probing pocket depth (PPD) was also assessed from the bottom of the sulcus to the gingival/mucosal margin using a plastic Michigan periodontal probe at four points around each unit at overdenture insertion at 0 (T0), 6 (T1), 12 (T2), and 36 (T3) months after insertion.

The bone levels were assessed from the first bone to the implant contact and the implant shoulder. Peri-implant bone resorption was assessed based on periapical radiographs taken at the time of implant surgery and at 3, 12, and 36 months after insertion. Baseline definition for bone loss calculations and aligned it with Albrektsson’s success criteria (bone loss from loading). Intraoral radiographs were produced using a paralleling technique and a film holder (XCP Instrument; Dentsply Rinn, York, PA, USA). The defined distance from one implant thread to the other was used for the calibration of the standardized measurements on the radiographic images. Cylindrical implants with standardized and quality-controlled manufacturing dimensions were ideal for calibration using radiographs. All implants were used to assess bone changes.

For immunological sampling and analysis, the deepest probing pocket depth around each implant was used for crevicular fluid sampling using sterile paper points with color-coded ends (VDW, 29 mm, ISO 25, Taper 02). Cytokines were detected using ELISA kits per the manufacturer’s instructions. BD OptEIA antibody pairs were used to measure IL-1β, IL-6, and TNFα (BD Pharmingen). ELISA data were quantified using an Ultra384 ELISA reader (Tecan, IBL International GmbH, Hamburg, Germany). At each session (at overdenture insertion (T0), 6 (T1), and 12 (T2) months after insertion), samples were taken from the same unit side. Before assessment, the sterile paper point was cut off at the lowest colored end under sterile conditions.

After sampling the peri-implant (crevicular) fluid up to the second colored end, the defined tip between the two marks was cut. Four of the tips were stored in one tube (Eppendorf, VWR International GmbH, 1.5 mL) filled with 350 µL phosphate-buffered saline (PBS, Sigma-Aldrich) and + 10% fetal calf serum (FCS, PAA Germany) at −80 °C. On each assessment day, a control sample consisting of four defined paper point tips was added that had absorbed a standard cytokine solution of known concentration and was stored in tubes filled with 350 µL PBS + 10% FCS. After the sample assessment, a calibration curve for the recovery of the ELISA-standard paper points was prepared. The lowest standard parameters for each value were assessed and projected to the dilution of 350 µL PBS + 10% FCS.

Prosthetic complications of the attachments (locator wear, fracture, and replacement) were evaluated. Each patient’s satisfaction was also measured using a standardized questionnaire for every single implant. Patients were asked to grade the complete restoration from 10 (perfect result, no complaints at all) to 1 (very poor) for pain, comfort, appearance, function, stability, cleaning, and satisfaction.

### 2.2. Statistical Analysis

The sample size was calculated a priori using G*Power software (Version 3.1.9.2, Düsseldorf, Germany [18,19]) based on the predefined primary inflammatory endpoint within the split-mouth design. The matched-pairs *t*-test model was used for estimation at the patient level. Although bleeding-on-probing (BOP) is an ordinal parameter, aggregated paired differences were treated as approximately continuous for planning purposes, which represents a commonly applied approach in clinical split-mouth studies when effect sizes are derived from comparable prior data. Based on a significance level of 0.05, an expected paired mean difference of 0.35 with a standard deviation of 0.5 [20], corresponding to an effect size of 0.71, and a statistical power of 80%, 18 participants were required. To account for a potential dropout rate of 8%, the sample size was increased to 20 participants. Secondary analyses were considered exploratory.

The patient served as the statistical unit in accordance with the split-mouth design. Continuous variables (e.g., marginal bone loss and cytokine concentrations) were analyzed using paired statistical tests after assessment of normal distribution. Repeated measures over time were evaluated using within-subject comparisons. Categorical outcomes, including implant survival and success rates, were analyzed descriptively and at the patient level. All analyses were conducted on a per-protocol (complete-case) basis. Missing data were not imputed.

## 3. Results

Ultimately, 18 patients attended the 3-year follow-up, as 2 patients refused to appear at the clinic. A total of 72 implants (36 titanium and 36 zirconia implants; 9 men and 9 women, with a mean age of 54, 3 years) were included. One patient with a zirconia implant dropped out due to insufficient cleaning and peri-implantitis. The overall implant survival rate after 3 years was 98.61%, and the overall success rate was 84.72%. The survival rate of the zirconia implants was 97.22%, and the success rate was 77.78%. The survival rate for the titanium implants was 100%, and the success rate was 91.70%.

All statistical analyses were performed on a per-protocol basis. Participants who did not complete the respective follow-up visit were excluded from the analysis of that time point. Two patients did not attend the 36-month follow-up examination; therefore, all data from these participants were excluded from the final 3-year analysis. No imputation or replacement of missing values was performed. Analyses were conducted using complete-case data only. The potential influence of excluding dropout cases on the interpretation of long-term outcomes is acknowledged as a limitation of the study.

**Figure 5:**
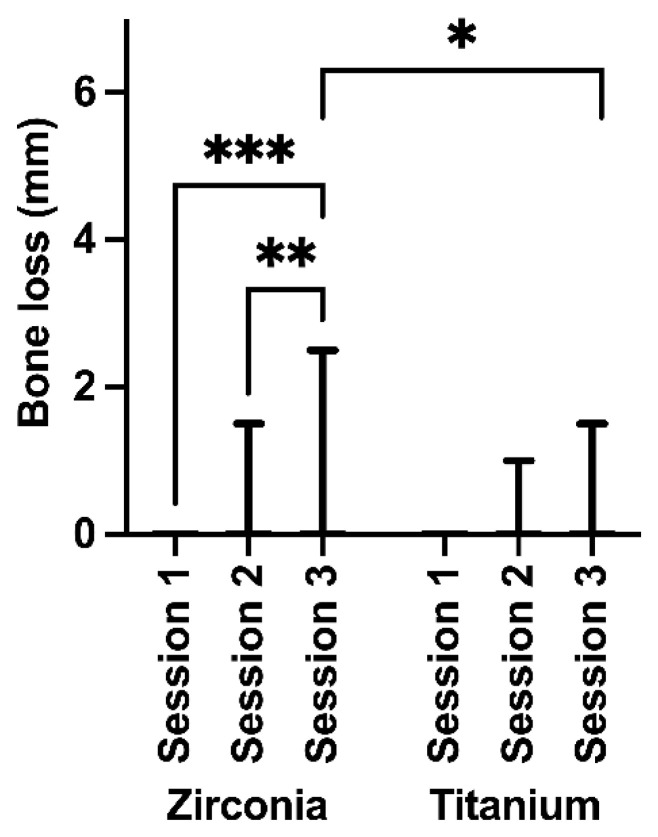
Evaluation of bone loss around the implants at 3 months (session 1), 12 months (session 2), and 36 months (session 3) after insertion. * = *p* ≤ 0.05; ** = *p* ≤ 0.01; *** = *p* ≤ 0.001.

Bone loss evaluation showed a significant increase in the zirconia group as the study progressed to the 3-year follow-up (*p* < 0.01), whereas the titanium group showed no significant bone loss over time. Comparison of both implant materials revealed a significantly greater bone loss for zirconia implants than for titanium implants at the end of the study (*p* value = 0.01, Figure 5). The lower success rate of zirconia implants was mainly due to marginal bone loss. Six zirconia implants exceeded the marginal bone loss threshold. After 3 years of functional loading, marginal bone loss was greater around zirconia implants compared with titanium implants. The mean bone loss in the zirconia group was 0.1190 mm (SD 0.1539), whereas titanium implants demonstrated a mean bone loss of 0.0463 mm (SD 0.0424).

**Figure 6:**
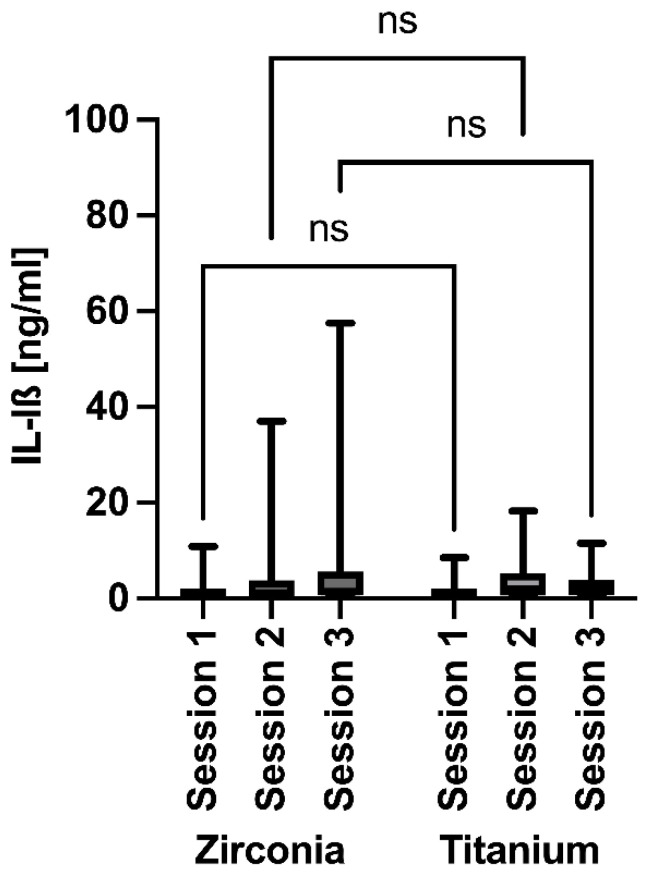
Measurements of IL-1β around the implants at 3 months (session 1), 6 months (session 2), and 12 months (session 3) after insertion. ns = non significant.

Immunological analysis indicated no significant differences in IL-1β levels within and between the implant groups over time, although the values increased more for the zirconia implants than for the titanium implants (Figure 6). For IL-1β, values ranged from 1.899 to 4.774 in the zirconia group and from 1.718 to 3.508 in the titanium group (means: 3.512 ± 1.469 vs. 2.725 ± 0.916; SEM: 0.848 vs. 0.529).

**Figure 7:**
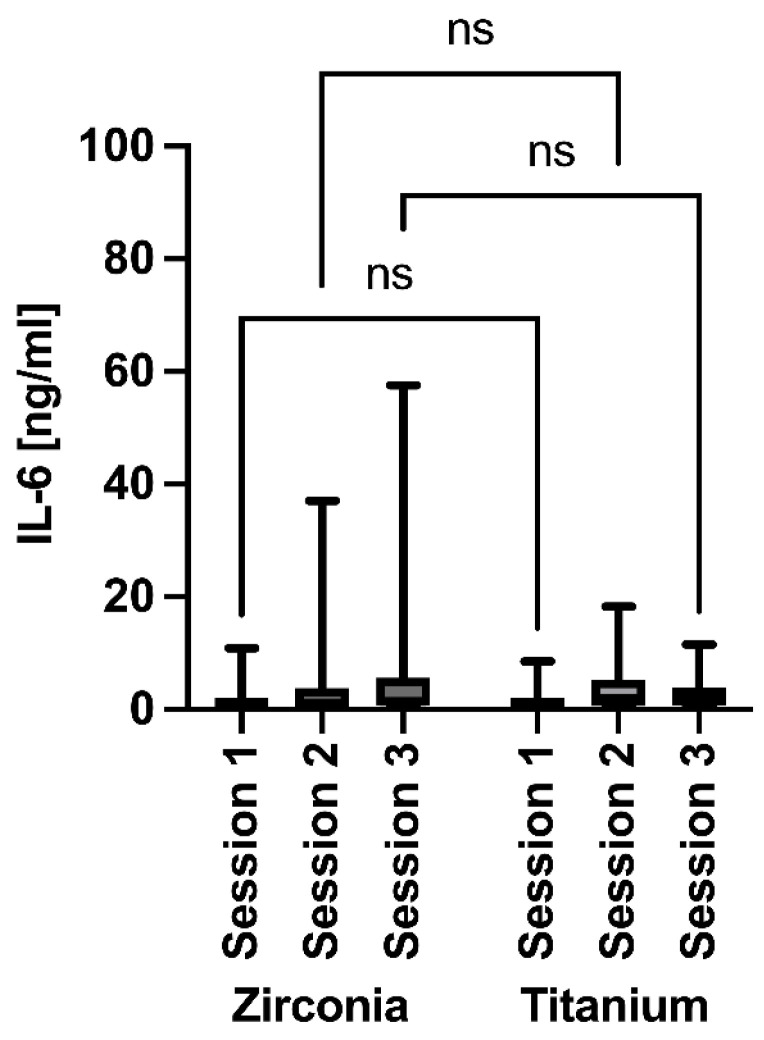
Evaluation of IL-6 around the implants at 3 months (session 1), 6 months (session 2), and 12 months (session 3) after insertion. ns = non significant.

A similar picture emerged for the IL-6 values, as no significant differences were detected within and between the implant groups over time (Figure 7). For IL-6, zirconia values ranged from 0.8500 to 1.100 and titanium values from 0.7625 to 0.8526 (means: 0.963 ± 0.127 vs. 0.806 ± 0.045).

**Figure 8:**
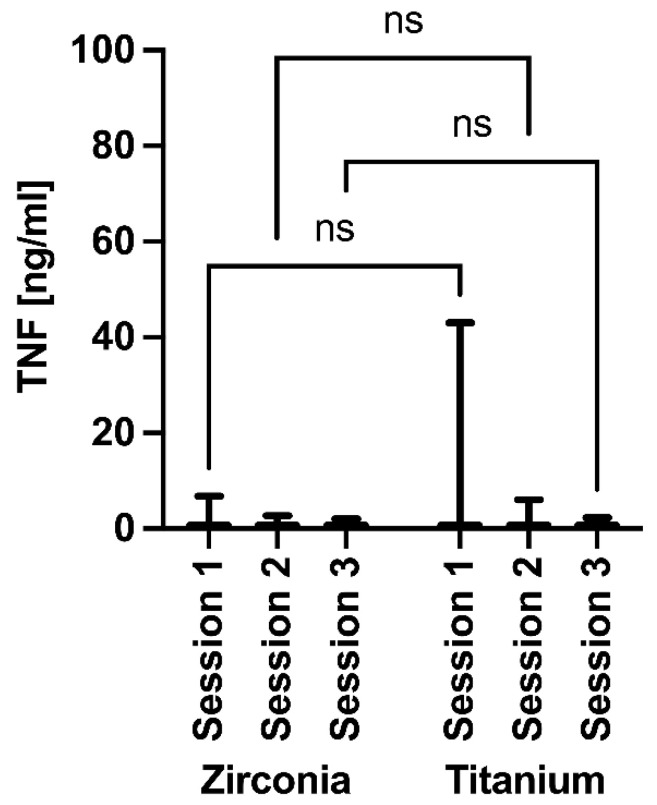
Assessment of TNFα around the implants at 3 months (session 1), 6 months (session 2), and 12 months (session 3) after insertion. ns = non significant.

The TNFα values showed slight declines for both implant materials during the follow-up, but again, no significant differences were observed within or between the implant groups over time (Figure 8). For TNF-α, zirconia ranged from 0.7444 to 0.9658 and titanium from 0.7447 to 1.758 (means: 0.860 ± 0.111 vs. 1.201 ± 0.514; SEM: 0.064 vs. 0.297).

When the patients were asked to grade the complete restoration from 10 (perfect result, no complaints at all) to 1 (very low result) at the 1-year and 3-year follow-ups (Table 1), they reported a high level of satisfaction at 1 year. However, the evaluation of the titanium implants and their aesthetic appearance was slightly reduced, whereas the zirconia implants performed slightly better.

**Figure 9:**
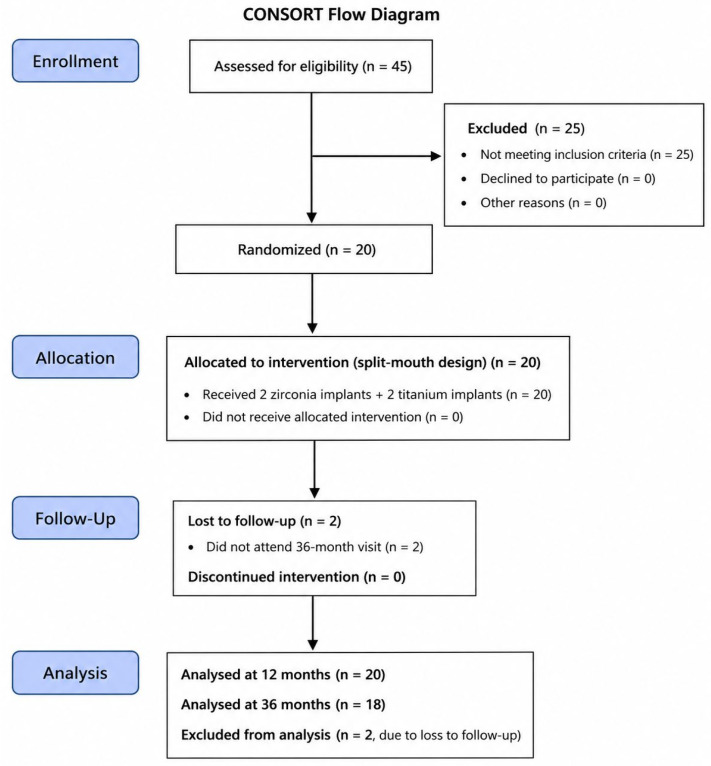
CONSORT flow diagram illustrating patient enrollment, allocation in the split-mouth design, follow-up, and analysis.

The functional outcomes after one year were graded lower for the titanium implants, indicating a slight disadvantage compared with the zirconia implants. Patients also indicated that the titanium implants were more difficult to clean. At the 3-year follow-up, the results remained largely consistent, but functional performance showed improvement. Patients also rated both the stability and the ease of cleaning more positively. However, their overall satisfaction had declined somewhat after three years. No locator fractures or replacements were recorded, and locator insert renewal was required in 5% of the cases at 3 years. This study was conducted and reported in accordance with the Consolidated Standards of Reporting Trials (CONSORT) guidelines (Figure 9).

## 4. Discussion

This 3-year split-mouth trial of mandibular overdenture rehabilitation revealed an overall implant survival rate of 98.61% and a success rate of 84.72%, in line with contemporary standards for both zirconia and titanium implants in edentulous jaws. Breaking this down, the zirconia group achieved a 97.22% survival and a 77.78% success rate, whereas the titanium group achieved a 100% survival rate and a 91.70% success rate. The lower success rate in the zirconia cohort merits detailed consideration and contextualization within the literature [21]. Customized computer-aided design/computer-aided manufacturing (CAD/CAM) titanium abutments may offer advantages over prefabricated stock abutments [22].

The 77.78% success rate observed for zirconia implants at 3 years is below commonly accepted clinical benchmarks for implant-supported overdentures and indicates reduced predictability in this indication compared with titanium implants. The reduced success rate observed for zirconia implants in the present overdenture setting suggests that stricter case selection criteria and careful long-term monitoring may be necessary when these implants are used under removable overdenture loading conditions.

Importantly, these findings should not be interpreted as a general inferiority of zirconia implants. In fixed prosthetic indications, such as single-tooth or short-span restorations without removable components, zirconia implants have demonstrated survival and success rates comparable to titanium implants.

Systematic reviews have reported that zirconia implants are a promising alternative to titanium, but they currently show a slightly higher risk of failure or complications. Abreu et al. concluded that zirconia survival is satisfactory in the short term, but long-term data beyond 5 years are insufficient [23]. Similarly, the RCT-only meta-analysis by Duan et al. indicated that survival was equivalent, but the success criteria were more reliably met by titanium [22]. Our findings confirm these patterns, as we had excellent survival in both groups but higher success rates with titanium.

In our study, zirconia implants exhibited significantly greater bone loss over 3 years, while bone loss with titanium implants remained stable. This is consistent with the findings from a randomized clinical trial by de Beus et al., who reported similar 1-year marginal bone levels but highlighted the need for longer follow-up to evaluate stability [24]. Another review has also noted that zirconia may be associated with higher marginal bone loss in specific settings, such as overdentures [25]. Shrivastava et al. emphasized that peri-implantitis and marginal bone loss results are “diversified” and sometimes favor titanium [26]. Thus, our results reinforce the perception that titanium remains a slightly more predictable material in terms of peri-implant bone stability.

We found no significant differences in the IL-1β, IL-6, or TNFα values over time or between the groups. This is in line with clinical data showing comparable cytokine profiles around zirconia and titanium implants when hygiene is adequate [27]. Clever et al., in an experimental peri-implant mucositis model, similarly reported no clear material-dependent differences in inflammation between zirconia and titanium [28]. Although some in vitro studies suggest that zirconia surfaces accumulate less biofilm [29], these microbiological advantages may not translate into measurable cytokine changes under stable clinical conditions. The absence of significant cytokine differences despite greater bone loss suggests that marginal bone changes may not be exclusively driven by classical inflammatory mechanisms but could also reflect biomechanical factors, load distribution, or implant–abutment interface characteristics. When interpreting the peri-implant cytokine data, it is important to consider the biological context of these measurements. Although cytokines were collected locally from peri-implant crevicular fluid, their expression is not exclusively site-specific and can be influenced by systemic host-related factors such as immune status, metabolic conditions, and individual inflammatory responsiveness. The split-mouth design used in this study reduces interindividual variability by allowing each patient to serve as their own control, thereby minimizing confounding by systemic differences between subjects. However, this approach cannot completely eliminate the influence of patient-level systemic factors on local inflammatory mediator expression. Consequently, the absence of significant differences in IL-1β, IL-6, and TNFα between zirconia and titanium implants should be interpreted with caution, as systemic host modulation may partially overshadow subtle material-related local effects.

Both implant types achieved high patient satisfaction, with zirconia favored slightly for esthetics and titanium for functional aspects. De Beus et al. reported similar findings, with zirconia implants showing favorable esthetic scores, while titanium implants maintained strong functional outcomes [24]. Alqahtani et al., in an overview of systematic reviews, concluded that zirconia is particularly advantageous in the anterior region for esthetics, while titanium retains mechanical superiority [30]. Our findings support this dichotomy, as zirconia excelled in esthetic perception and titanium in predictability.

Locator stability was excellent, with no fractures occurring and only 5% of the locators requiring renewal over the three years of the study. These findings are consistent with systematic reviews reporting low complication rates for locator attachments [31,32]. Thus, the prosthetic design and attachment selection appear more critical than the implant material itself in determining the prosthetic maintenance burden. The snap-fit connection of the Novaloc/Pureloc^®^ (Straumann GmbH, Freiburg, Germany) matrix to the abutment or locator male is based on the locking mechanism of the retention insert across the functional range of the Novaloc/Pureloc^®^ abutment or the locator male. The construction of the retention insert and the use of PEEK as a material enable the Novaloc/Pureloc^®^ matrix to cater to several extremely divergent abutment positions without wear and tear on the retention inserts. In this study, no problems occurred with the implant axis. The dilation area arranged between the matrix housing and the retention insert allows the retention insert to expand without any strain, thereby significantly extending the insert’s lifespan. Titanium implants remain the most predictable for overdenture support, especially regarding marginal bone stability and strict success criteria. Zirconia implants are a valid option, particularly for patients concerned with esthetics or metal-free rehabilitation, but these implants require careful monitoring. Our findings suggest that material choice does not significantly affect local cytokine responses, thereby underlining the importance of patient risk factors and hygiene as decisive factors regulating peri-implant inflammation. PROM findings highlight a clinically relevant trade-off: zirconia was perceived as advantageous in esthetics and cleaning, whereas titanium demonstrated superior stability and long-term predictability. Regarding PROM data and material distinction, patients were instructed and clinically guided to evaluate each implant side separately during follow-up visits. As part of the split-mouth design, the overdenture was retained bilaterally, and patients were asked to compare their subjective perception of each side (e.g., stability, cleaning, esthetics) during function and hygiene procedures. We acknowledge that patients experience the prosthesis as a single restoration and that side-specific differentiation may therefore be limited. Limitations of this study include the relatively small patient-level sample size, the clustered split-mouth design, limited immunological follow-up to 12 months, cytokine endpoint power, absence of blinding, and restricted generalizability to other implant systems or prosthetic designs. The study’s follow-up period, prosthetic modality (mandibular overdentures), and sample size limit its generalizability. Longer-term studies (>5–10 years) are needed to clarify whether the marginal bone loss differences widen with time. The retrospective registration of the trial should also be acknowledged as a limitation. Even though the study protocol and endpoints were reported as unchanged after study initiation, retrospective registration reduces methodological transparency because it does not allow full independent verification that outcomes, hierarchy of endpoints, and analytical priorities were prospectively defined before patient enrollment. This may also increase the perceived risk of selective outcome emphasis in the final report.

Future randomized controlled studies should stratify the implants by prosthetic type, implant surface, and bone quality and should incorporate longitudinal microbiologic and immunologic analyses.

## 5. Conclusions

Titanium implants demonstrated higher success rates and superior marginal bone stability compared with zirconia implants under overdenture loading conditions. While survival was high in both groups, zirconia implants showed clinically relevant reductions in success and increased marginal bone loss, necessitating stricter case selection and long-term monitoring.

## Figures and Tables

**Table 1:** Patients were asked to grade the complete restoration at follow-ups using a questionnaire with a scoring range from 10 (perfect result, no complaints at all) to 1 (very poor result).

	1-Year Follow-Up (Mean)		3-Year Follow-Up (Mean)	
	Titanium	Zirconia	Titanium	Zirconia
**Pain**	10	10	9.8	9.8
**Comfort**	9.9	10	10	10
**Appearance**	8.5	9.8	9.3	10
**Function**	8	9	9.2	9.3
**Stability**	8.6	8.9	10	9.1
**Cleaning**	8.0	9.1	8.6	9.3
**Satisfaction**	10	10	9.4	9.5

## Data Availability

The original contributions presented in this study are included in the article. Further inquiries can be directed to the corresponding author.

## References

[1] Cionca N., Hashim D., Mombelli A. (2017). Zirconia dental implants: Where are we now, and where are we heading?. Periodontology 2000.

[2] Chappa N., Valaparla J., Gadde H., Venkatapathi A., Sree H.V., Srija S.C., Parthiban S. (2025). Metal-Free Dental Implants: A Narrative Review of Zirconia and Emerging Alternative Materials. J. Pharm. Bioallied Sci..

[3] Abdurakhmon K., Wu J., Li S., Wei C. (2025). Biomechanical evaluation of implant materials and connection designs: A structured narrative review of titanium and zirconia. Odontology.

[4] Andreiotelli M., Kohal R.J. (2009). Fracture strength of zirconia implants after artificial aging. Clin. Implant. Dent. Relat. Res..

[5] Rocchietta I., Fontana F., Addis A., Schupbach P., Simion M. (2009). Surface-modified zirconia implants: Tissue response in rabbits. Clin. Oral Implant. Res..

[6] James J.R., Kharat A., Chinnakutti S., Kamble S., Mandal M., Das A. (2025). The Future of Dental Implants: A Narrative Review of Trends, Technologies, and Patient Considerations. Cureus.

[7] Neugebauer J., Schoenbaum T.R., Pi-Anfruns J., Yang M., Lander B., Blatz M.B., Fiorellini J.P. (2023). Ceramic Dental Implants: A Systematic Review and Meta-analysis. Int. J. Oral Maxillofac. Implant..

[8] de Medeiros R.A., Vechiato-Filho A.J., Pellizzer E.P., Mazaro J.V., dos Santos D.M., Goiato M.C. (2013). Analysis of the peri-implant soft tissues in contact with zirconia abutments: An evidence-based literature review. J. Contemp. Dent. Pract..

[9] Kniha K., Schlegel K.A., Kniha H., Modabber A., Neukam F., Kniha K. (2019). Papilla-Crown Height Dimensions around Zirconium Dioxide Implants in the Esthetic Area: A 3-Year Follow-Up Study. J. Prosthodont..

[10] Welander M., Abrahamsson I., Berglundh T. (2008). The mucosal barrier at implant abutments of different materials. Clin. Oral Implant. Res..

[11] Nascimento C.D., Pita M.S., Fernandes F., Pedrazzi V., de Albuquerque Junior R.F., Ribeiro R.F. (2014). Bacterial adhesion on the titanium and zirconia abutment surfaces. Clin. Oral Implant. Res..

[12] Alves A.P., Monteiro C.G.J., Silva A.M.O., Louro R.S., da Silva A.M.P., Moraschini V. (2026). Clinical performance of immediately loaded zirconia implants: A systematic review with meta-analysis. J. Dent..

[13] Kniha K., Gahlert M., Hicklin S., Bragger U., Kniha H., Milz S. (2016). Evaluation of Hard and Soft Tissue Dimensions Around Zirconium Oxide Implant-Supported Crowns: A 1-Year Retrospective Study. J. Periodontol..

[14] Albrektsson T., Zarb G., Worthington P., Eriksson A.R. (1986). The long-term efficacy of currently used dental implants: A review and proposed criteria of success. Int. J. Oral Maxillofac. Implant..

[15] Mombelli A., van Oosten M.A., Schurch E., Land N.P. (1987). The microbiota associated with successful or failing osseointegrated titanium implants. Oral Microbiol. Immunol..

[16] Tonetti M.S., Schmid J. (1994). Pathogenesis of implant failures. Periodontology 2000.

[17] Bragger U., Hugel-Pisoni C., Burgin W., Buser D., Lang N.P. (1996). Correlations between radiographic, clinical and mobility parameters after loading of oral implants with fixed partial dentures. A 2-year longitudinal study. Clin. Oral Implant. Res..

[18] Faul F., Erdfelder E., Buchner A., Lang A.G. (2009). Statistical power analyses using G*Power 3.1: Tests for correlation and regression analyses. Behav. Res. Methods.

[19] Faul F., Erdfelder E., Lang A.G., Buchner A. (2007). G*Power 3: A flexible statistical power analysis program for the social, behavioral, and biomedical sciences. Behav. Res. Methods.

[20] Kniha K., Milz S., Kniha H., Ayoub N., Holzle F., Modabber A. (2018). Peri-implant Crestal Bone Changes Around Zirconia Implants in Periodontally Healthy and Compromised Patients. Int. J. Oral Maxillofac. Implant..

[21] Robaian A., Hamed M.M., Ahmed Y., Hassanein F.E.A. (2025). Comparative Evaluation of Customized CAD/CAM vs. Stock Titanium Abutments for Immediate Implant Placement in Class II Extraction Sockets: A Randomized Controlled Trial. Dent. J..

[22] Duan C., Ye L., Zhang M., Yang L., Li C., Pan J., Wu Y., Cao Y. (2023). Clinical performance of zirconium implants compared to titanium implants: A systematic review and meta-analysis of randomized controlled trials. PeerJ.

[23] Abreu F., Correia F., Caetano T., Faria-Almeida R. (2025). The Survival Rate of Zirconia Versus Titanium Dental Implants: A Systematic Review. Surgeries.

[24] de Beus J.H.W., Cune M.S., Slot J.W.A., Jensen-Louwerse C., la Bastide-van Gemert S., Meijer H.J.A., Raghoebar G.M., Schepke U. (2024). A randomized clinical trial on zirconia versus titanium implants in maxillary single tooth replacement. Clin. Oral Implant. Res..

[25] Padhye N.M., Calciolari E., Zuercher A.N., Tagliaferri S., Donos N. (2023). Survival and success of zirconia compared with titanium implants: A systematic review and meta-analysis. Clin. Oral Investig..

[26] Shrivastava D., Quadri S.A., Alshadidi A.A.F., Saini R., Dewan M., Fernandes G.V.O., Srivastava K.C. (2025). Clinical Assessment of the Relationship of Dental Implant Materials (Titanium and Zirconia) and Peri-Implantitis: A Systematic Review. J. Maxillofac. Oral Surg..

[27] Cionca N., Hashim D., Cancela J., Giannopoulou C., Mombelli A. (2016). Pro-inflammatory cytokines at zirconia implants and teeth. A cross-sectional assessment. Clin. Oral Investig..

[28] Clever K., Schlegel K.A., Kniha H., Conrads G., Rink L., Modabber A., Hölzle F., Kniha K. (2019). Experimental peri-implant mucositis around titanium and zirconia implants in comparison to a natural tooth: Part 1-host-derived immunological parameters. Int. J. Oral Maxillofac. Surg..

[29] Roehling S., Astasov-Frauenhoffer M., Hauser-Gerspach I., Braissant O., Woelfler H., Waltimo T., Kniha H., Gahlert M. (2017). In Vitro Biofilm Formation on Titanium and Zirconia Implant Surfaces. J. Periodontol..

[30] Alqahtani S.M., Chaturvedi S., Alkhurays M., Al Mansoori M.A., Mehta V., Chaturvedi M. (2025). Clinical effectiveness of Zirconia versus titanium dental implants in anterior region: An overview of systematic reviews. Eur. J. Med. Res..

[31] Prasad S., Faverani L.P., Santiago Junior J.F., Sukotjo C., Yuan J.C. (2024). Attachment systems for mandibular implant-supported overdentures: A systematic review and meta-analysis of randomized controlled trials. J. Prosthet. Dent..

[32] Chaware S.H., Thakkar S.T. (2020). A systematic review and meta-analysis of the attachments used in implant-supported overdentures. J. Indian Prosthodont. Soc..

